# Comparative Optical Response of DNA-Natural and Synthetic Food Colorant Systems

**DOI:** 10.3390/polym18182256

**Published:** 2026-09-16

**Authors:** Ana-Maria Manea-Saghin, Petronela Gheorghe, Jaroslaw Mysliwiec

**Affiliations:** 1 Research Center for Environmental Protection and Eco-Friendly Technologies, National University of Science and Technology POLITEHNICA of Bucharest, 1–7 Polizu Street, 011061 Bucharest, Romania; 2 National Institute for Laser, Plasma and Radiation Physics, 409 Atomistilor Street, 077125 Magurele, Romania; 3 Soft Mater Optics Group, Wroclaw University of Science and Technology, 27, 50-370 Wroclaw, Poland

**Keywords:** deoxyribonucleic acid (DNA), quinoline yellow (QY), sunset yellow (SY), riboflavin (RB), optical responsivity, pump–probe interferometry

## Abstract

Biopolymers functionalized with chromophores exhibit interesting optical properties with a high potential for applications in photonics. Quinoline yellow (QY), sunset yellow (SY) and riboflavin (RB) are commonly used as colorants in the food industry, which makes them suitable candidates for the development and manufacture of environmentally friendly devices for integrated photonics. The incorporation of these dyes into a deoxyribonucleic acid (DNA) matrix could significantly contribute to enhance the optical response and sensitivity of the obtained composites. This enhancement is mainly due to the interaction between the conjugated π-electrons from the aromatic groups of the chromophores and the DNA matrix. Also, it may influence the appearance of interesting molecular structural arrangements and modify the local electronic environment. The linear optical analysis was performed by UV-Vis, fluorescence and Fourier transform infrared (FTIR) spectroscopies. At the same time, the pump–probe interferometric method was used to study the light-induced refractive index modification.

## 1. Introduction

In recent decades, due to their interesting optical properties, natural polymers have received increasing interest, both scientific and applicative. Incorporation of vegetal-origin chromophores, dyes or synthetic colorants, into various biopolymers, contributes significantly to improving their photonic functionalities.

Quinoline yellow (QY) dye has the empirical formula C_18_H_9_NO_8_S_2_Na_2_, and consists of sodium salts of mono-, di-, and tri-sulfonated 2-(2-quinolyl) indan-1,3-dionedisulfonate. QY has aromatic rings that bring a richness in π-electrons for the manufacture of optoelectronic devices [1,2]. Quinoline derivatives are widely studied owing to their exceptional mechanical properties, which help create materials that are highly efficient in electron transport. Features like good film formation, high electron mobility, high photoluminescence efficiency, and high thermal stability make quinoline derivatives useful in organic light-emitting diodes (OLEDs). Therefore, compounds containing quinoline help improve their brightness and increase the luminescence efficiency [3,4]. A recent study reported fabrication and application of QY thin films in optoelectronics as a photodiode [1].

Sunset yellow (SY) is a mono-azo di-sulfonated hydroxy dye, known as disodium 2-hydroxy-1-(4-sulphonatophenylazo) naphthalene-6-sulphonate (C_16_H_10_N_2_Na_2_O_7_S_2_), with conjugated aromatic rings and an azo functional group. It is used as an additive in pharmaceutical tablets and capsules, as well as in confectionery products, drinks, and various other segments of the food industry [5,6,7].

Riboflavin (RB), also known as vitamin B2 (C_17_H_20_N_4_O_6_), is found in plants and animals, and is a sustainable source of material with a strong absorption band in the blue and UV spectra, which makes it effective in photochemical reactions. When irradiated, excited riboflavin can convert triplet oxygen into singlet oxygen. Excited riboflavin can catalyze various chemical transformations in organic synthesis, environmental chemistry, polymerizations, and biological systems for applications such as photodynamic therapy [8].

Deoxyribonucleic acid (DNA) was successfully used as a matrix for photonic applications [9,10,11,12]. The bases of deoxyribonucleic acid are π-conjugated materials with delocalized π-electrons, which makes them suitable as optical materials for electron transfer in photonic applications due to π–π* interband orbital transitions [13].

Optoelectronics is a versatile branch of photonics that has gained interest in recent decades. This is due, in particular, to the use of eco-friendly materials through the incorporation of natural colorants and synthetic dyes into matrices to enhance the optical response of interest. In this regard, organic compounds were extensively studied due to the presence of conjugated systems with π-electrons, which allows them to be widely employed for the manufacture of electronic and optoelectronic devices.

In this study, water-soluble quinoline yellow, sunset yellow and riboflavin were used as dyes for incorporation into the DNA matrix. The characterization of linear optical properties was realized using UV-Vis, fluorescence and infrared spectroscopies, which have been employed for the analysis of main bioactive compounds. On the other hand, their performances for application in optoelectronics were evaluated from a nonlinear optical response point of view by the pump–probe interferometric method [14,15,16].

**Figure 1:**
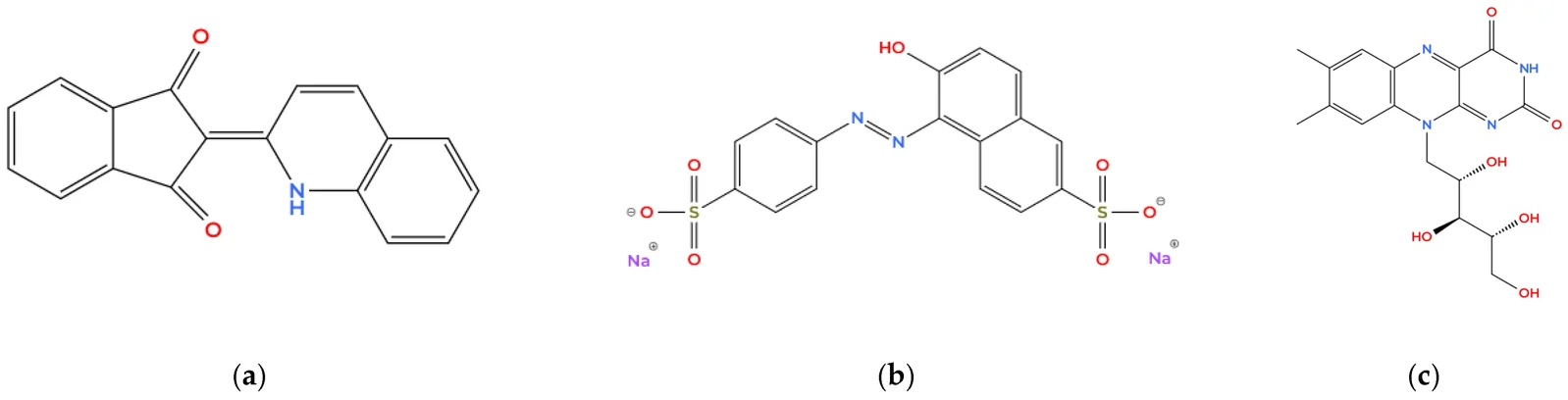
Chemical structures of (**a**) quinoline yellow, (**b**) sunset yellow, (**c**) riboflavin.

The chemical structures of the studied molecules—quinoline yellow, sunset yellow and riboflavin—are shown in Figure 1.

The results of this study will provide new ideas for material design and development of multi-functional eco-friendly and integrated photonic devices. These materials are important for realizing systems for control light by light.

## 2. Materials and Methods

### 2.1. Materials

The deoxyribonucleic acid (DNA) used in this study, with a purity of 96%, was acquired from the Chitose Institute of Science and Technology, Chitose, Japan, Hokkaido. The DNA was obtained from salmon sperm and came in solid form as an ivory-colored string. It is water-soluble only, allowing easy preparation of solutions for experimental use. Salmon sperm DNA is commonly used in various biochemical and biophysical studies due to its availability, relatively large molecular size, and well-characterized structure. The size and molecular weight of the DNA depend on factors such as the species origin and the developmental stage of the organism. Due to its biocompatibility and natural origin, salmon sperm DNA also finds applications in nanotechnology and the development of biopolymer-based materials. Its solubility in water facilitates its use in creating hydrogels, thin films, and other biomaterials with potential uses in drug delivery, biosensing, and tissue engineering [17].

Quinoline yellow in the form of an extra pure and water-soluble orange powder with a 477.38 g/mol molar mass, sunset yellow in the form of a red powder with a molecular mass of 452.37 g/mol and dye content of 90%, and riboflavin in the form of a yellow-orange powder with a molecular mass of 376.36 g/mol were purchased from Sigma-Aldrich, St. Louis, MO, USA.

Acetonitrile was purchased from Sigma-Aldrich and used for solubilization of riboflavin.

### 2.2. Preparation of Solutions

The QY, SY and RB solutions were prepared as stock solutions with a concentration of 10^−3^ M. QY and SY were prepared in distilled water, while RB was prepared in a 1:1 mixture of ACN: H_2_O. Next, a DNA solution having a concentration of 0.5 g/L was doped with QY, SY and RB using the molar concentrations given in Table 1. The diluted solutions were prepared from the stock solutions.

### 2.3. Spectroscopic Characterization by UV-Vis

UV-Vis absorption spectra of the prepared solutions were recorded using a Jasco V-570 UV-Vis spectrophotometer (JASCO Corporation, Tokyo, Japan) over the wavelength range of 200–600 nm. The instrument settings were: bandwidth 0.2 nm, scanning speed of 400 nm/min, and data pitch of 1.0 nm. Measurements were performed using a silica cuvette with a 1 cm path length. Each sample was measured at room temperature under identical conditions. Before measuring the samples, a baseline spectrum was recorded to ensure accuracy.

### 2.4. Photoluminescence Characterization

Fluorescence spectra were recorded using a Jasco FP-6500 spectrofluorometer from JASCO Corporation, Tokyo, Japan with excitation and emission bandwidths of 10 nm each and a scan speed of 1000 nm/min. Measurements were performed in a transparent silica cuvette with a 1 cm path length. The samples were excited at various wavelengths, and emission spectra were collected over the corresponding ranges. All measurements were carried out at room temperature. Samples were prepared at different concentrations.

### 2.5. Fourier Transform Infrared (FTIR) Spectroscopy

Attenuated total reflection Fourier transform infrared (ATR-FTIR) spectroscopy was performed using a Thermo Nicolet 6700 FTIR spectrophotometer from Thermo Electron Corporation, Madison, WI, USA. Solution samples were directly applied to the ATR crystal. Spectra were collected over the range of 4000 to 400 cm^−1^ at room temperature with a spectral resolution of 6 cm^−1^. Each spectrum was obtained by averaging 64 scans to improve the signal-to-noise ratio. A background spectrum was recorded prior to sample analysis to compensate for atmospheric interference. Data processing included baseline correction and normalization.

### 2.6. Pump–Probe Interferometry

**Figure 2:**
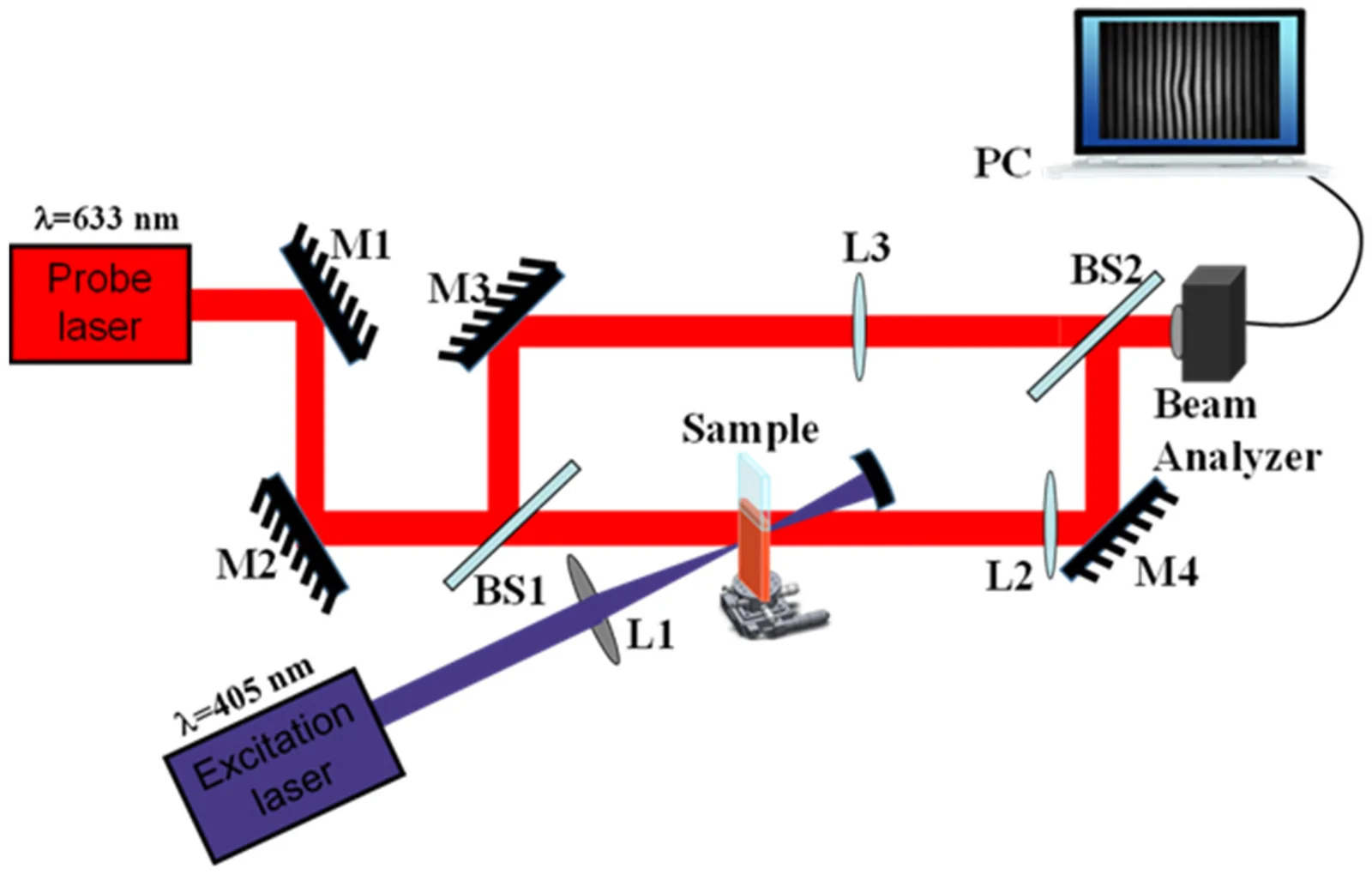
Schematic presentation of the experimental setup used for the study of light-induced phase modulation study.

A pump–probe interferometric method was employed to investigate the light-induced phase modulation and determine the nonlinear refractive index of the investigated solutions. The experimental setup is based on a Mach–Zehnder interferometer, as described in detail in [16]. It is schematically shown in Figure 2.

A He–Ne laser beam (λ = 633 nm) was divided into two beams of approximately equal intensity by the beam splitter BS1, forming the test and reference arms of the interferometer. Mirrors M1 and M2 were used for precise alignment of the He–Ne beam with respect to BS1. The test beam, hereafter referred to as the probe beam, propagated through the investigated solution, whereas the reference beam propagated through free space. The intensity of the He–Ne probe beam was of the order of a few mW. Its wavelength does not affect the optical response in the investigated samples, as it was outside their sensitivity range and sufficiently far from the corresponding absorption bands. This was confirmed by UV-Vis absorption spectroscopy measurements.

The probe beams propagating through the test and reference arms were subsequently directed by mirrors M3 and M4, respectively, and recombined by the beam splitter BS2 at a small angle. The resulting interference pattern was recorded on the photosensitive matrix of a laser beam analyzer (LaserCam-HR, Beam Diagnostics Digital CMOS Camera, Coherent, Saxonburg, PA, USA; LBA). The two beams generated a system of approximately parallel and equidistant interference fringes. The spatial period of the fringes was determined by the angle between the two interfering beams. The investigated solution was placed in the test arm of the interferometer using a removable optical-quality quartz cuvette with an optical path length of 0.5 mm and a volume of 130 μL. The cuvette was mounted on an (x,y,z) translation stage, allowing precise and reproducible positioning of the sample within the interferometric setup.

A pump beam with an appropriate wavelength (λ = 405 nm, LAB 405-1000 diode laser, Laser TACK GmbH, Germany, Kassel), selected within the sensitive spectral range of the investigated sample, close to the absorption peak as confirmed by UV-Vis spectroscopy measurements, is focused by lens L1, onto the sample. The pump beam locally excites the nonlinear optical response of the sample.

## 3. Results and Discussion

### 3.1. UV-Vis Characterization

**Figure 3:**
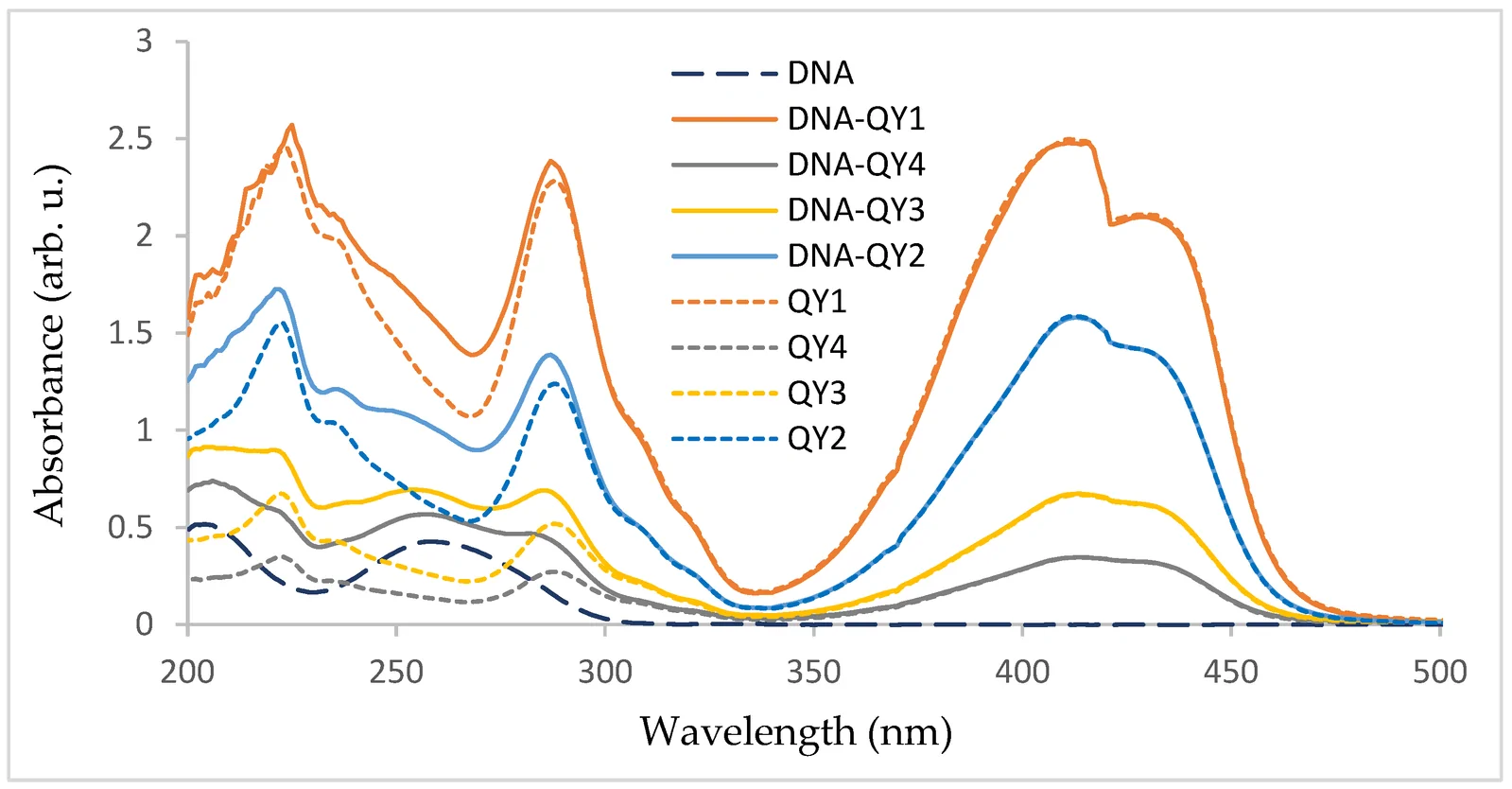
UV-Vis absorption spectra of DNA, QY and DNA-QY solutions at different QY concentrations.

The absorption spectrum of DNA obtained from salmon sperm solution shows an absorption maximum at wavelengths below 225 nm, which represents a sum of the absorptions originating from the phosphate groups and deoxyribose fragments. The second absorption maximum is seen around 260 nm. It is due to electronic transitions of the aromatic bases of nucleic acids, suggesting a weak delocalization of π-electrons in the planes of DNA base pairs [18]. DNA bases are π-conjugated structures with delocalized π-electrons. Therefore, the presence of DNA bases in the structure gives DNA unique properties, allowing it to be used as an optical material for photonics applications due to the ease with which electronic transitions can be made from the π-bonding molecular orbital to the π* antibonding orbital [13]. The DNA solutions were prepared in double-distilled water, and from the UV-Vis spectra (Figure 3), a specific absorption maximum for DNA at 260 nm due to nucleic acids [19] can be seen, as well as an absorption maximum at 208 nm, which is attributed to the phosphate groups and deoxyribose fragments [18].

Due to their high photoluminescent properties, quinoline yellow and its derivatives are suitable materials for use in electron transport, specifically in creating OLED-type devices [20]. In the current study, quinoline yellow was dissolved in double-distilled water. In the study by dos Santos et al. [2], the maximum absorption for quinoline dissolved in ethanol was found at 272 nm. For the DMSO solvent, the maximum was noted at 274 nm [2]. It is important to mention that the absorption phenomenon depends on a number of factors that can cause shifts in the electronic structure of molecules, such as viscosity, polarity, and of course, the solubility of the compounds [2,20].

The π-π* and n-π* transitions are generally more prone to exhibiting shifts of the absorption maxima depending on the polarity of the solvent, both in terms of wavelength and spectrum intensity [2,20]. In the current study (Figure 3), for a concentration of 2 × 10^−5^ M, it is worth noting that the absorption maxima were identified at 224 nm, 289 nm, 413 nm, and 432 nm.

The π-π* transitions occur at wavelengths of 224/289 nm and are due to the delocalized π-electrons in the aromatic nuclei, while the transitions around 413/432 nm are attributed to intramolecular n-π* electronic transitions from the amino group on the aromatic nucleus [2].

Sunset yellow is a food coloring dye that belongs to the azo dye family [21].

**Figure 4:**
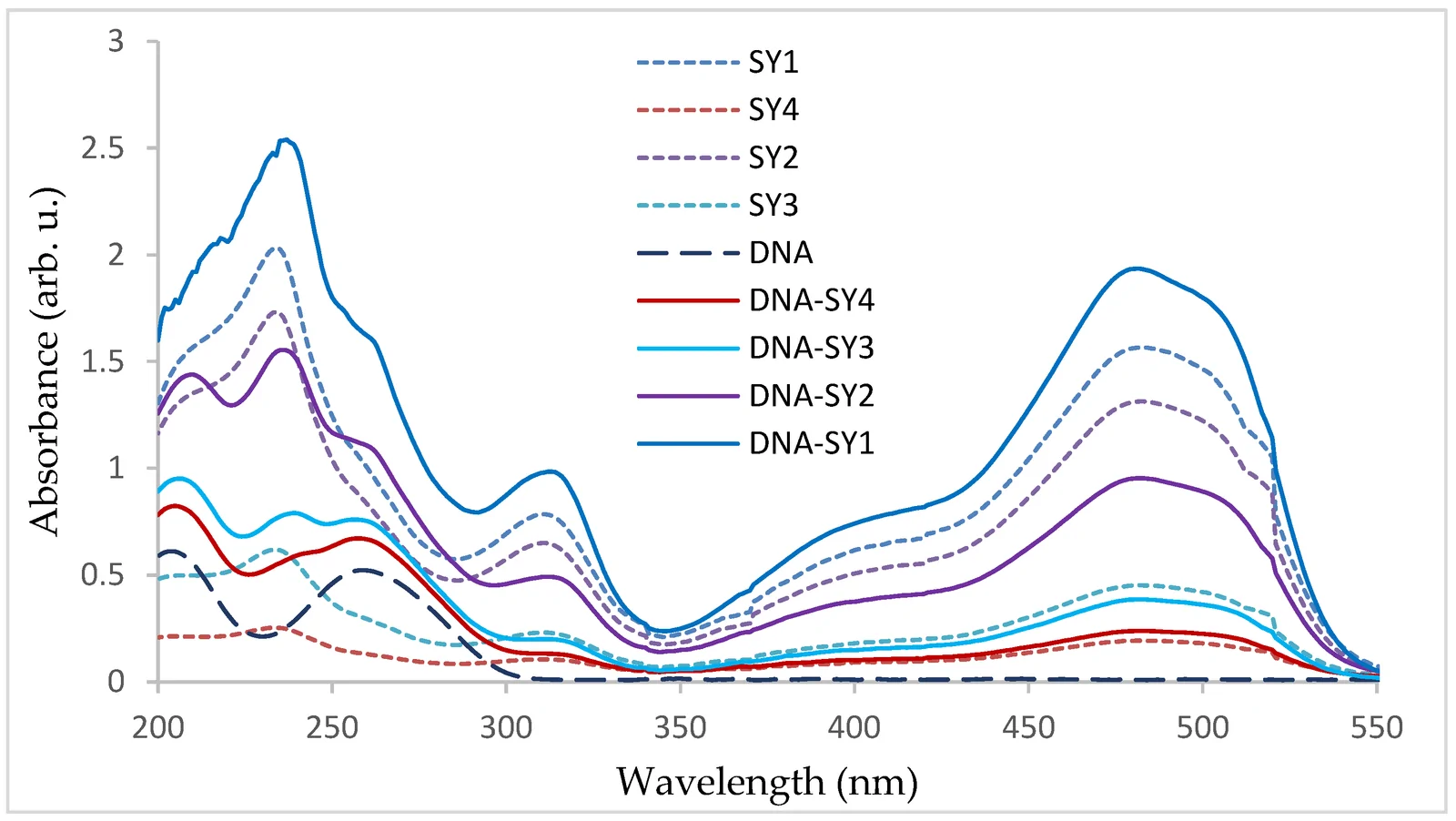
UV-Vis absorption spectra of DNA, SY and DNA-SY solutions at different SY concentrations.

The absorption maximum for a standard sunset yellow solution was identified in a study by Güray [22] to be 482 nm and 480 nm [23]. Considering the chemical structure of the dye, the two absorption bands observed at 315 nm and 480 nm are attributed to transitions in the aromatic nucleus and the azo bond [23]. In the current study (Figure 4), the absorption maxima were identified at 234/311/483 nm. The peak at 311 nm is attributed to the π-π* transitions of the substituted aromatic core in the structure, while the absorption peak at 483 nm is attributed to the azo bond. At the same time, the peak at 234 nm is attributed to π-π* transitions in the aromatic rings [23]. A significant increase in absorbance intensity is observed at the highest SY concentration incorporated into the DNA matrix, whereas the remaining SY concentrations exhibit a decrease in absorbance.

Riboflavin can be dissolved in different solvents, such as water, acetonitrile, or dimethyl sulfoxide, but the shifts due to the solvent polarity negligibly affect the first maximum in the visible range at around 450 nm, while the shifts are more pronounced for the absorption maximum at around 350 nm [24]. According to the literature data, riboflavin shows absorption bands in the UV range at wavelengths of 222, 266, and 373 nm, and in the visible range at 445 nm [25]. Therefore, in the study by Daidone et al. [24] of riboflavin dissolved in water, the absorption maximum observed at 365 nm experiences a hypsochromic effect and shifts to 350 nm in ACN and to 345 nm in DMSO [24]. According to [24], the absorption maximum observed at 444 nm in water shifts to 439 nm in ACN and to 448 nm in DMSO. Nevertheless, Coskun et al. [8] reported an absorption maximum of riboflavin at λ = 450 nm in DMSO.

**Figure 5:**
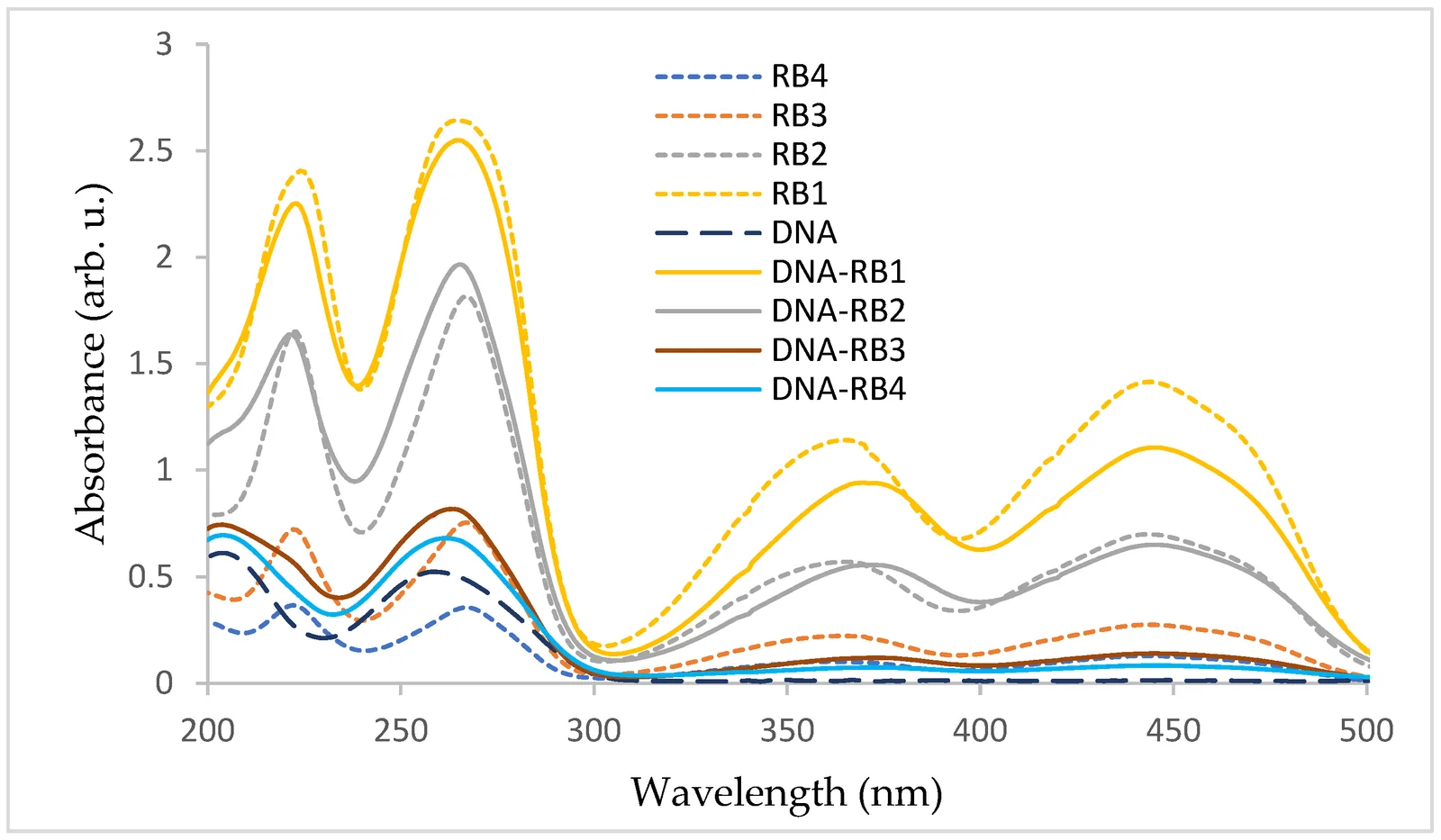
UV-Vis absorption spectra of DNA, RB and DNA-RB solutions at different RB concentrations.

In the present study (Figure 5), RB exhibits two doublets in the absorption spectrum: one located at 224–269 nm, and the second at 365–444 nm. The absorption maximum observed at 444 nm in the ACN: H_2_O (1:1) system is attributed to the n-π* transitions [26]. The absorption maxima seen at 222 nm (ACN: H_2_O), and at 266 nm (ACN: H_2_O) are attributed to π-π* transitions occurring in the aromatic rings of riboflavin [26].

### 3.2. Photoluminescence

**Figure 6:**
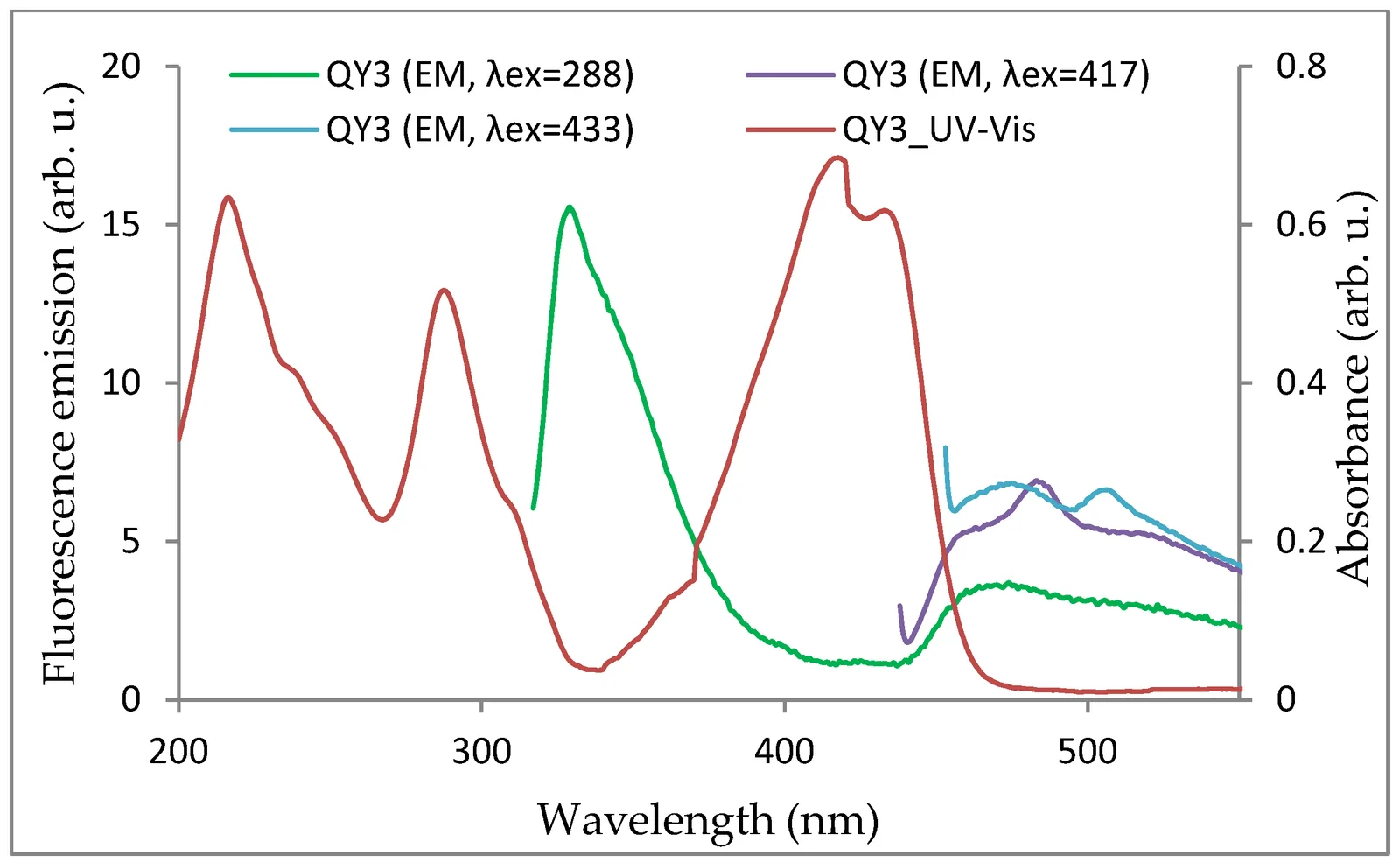
Photoluminescence spectra of QY solutions.

Figure 6 shows the emission spectra for QY. The Stokes shift was calculated as 62 nm (with emission at 479 nm and excitation at 417 nm). Therefore, QY is suitable for potential photonics applications due to the relatively large difference between the emission and the excitation spectra, which prevents the overlap of respective energy bands.

**Figure 7:**
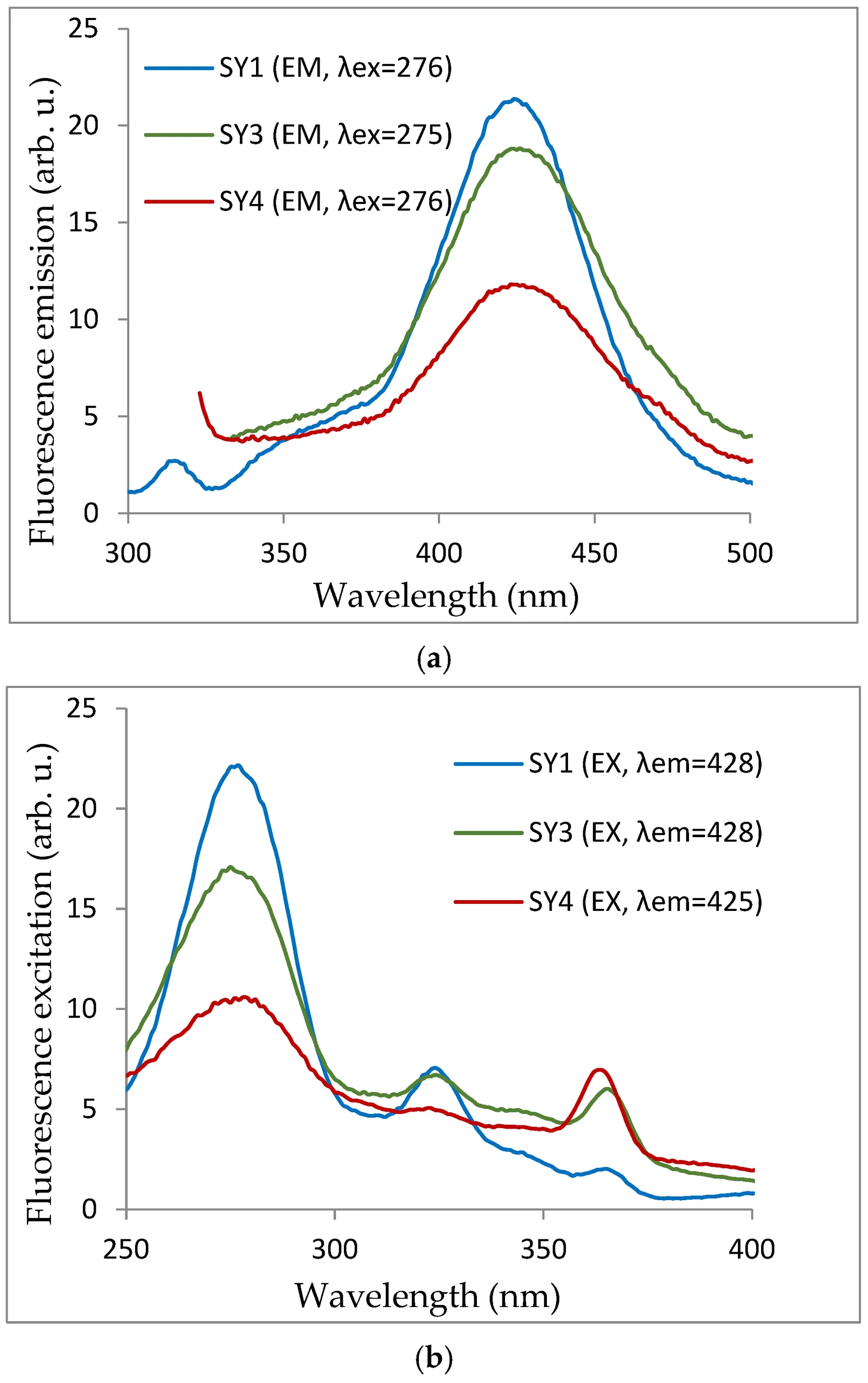

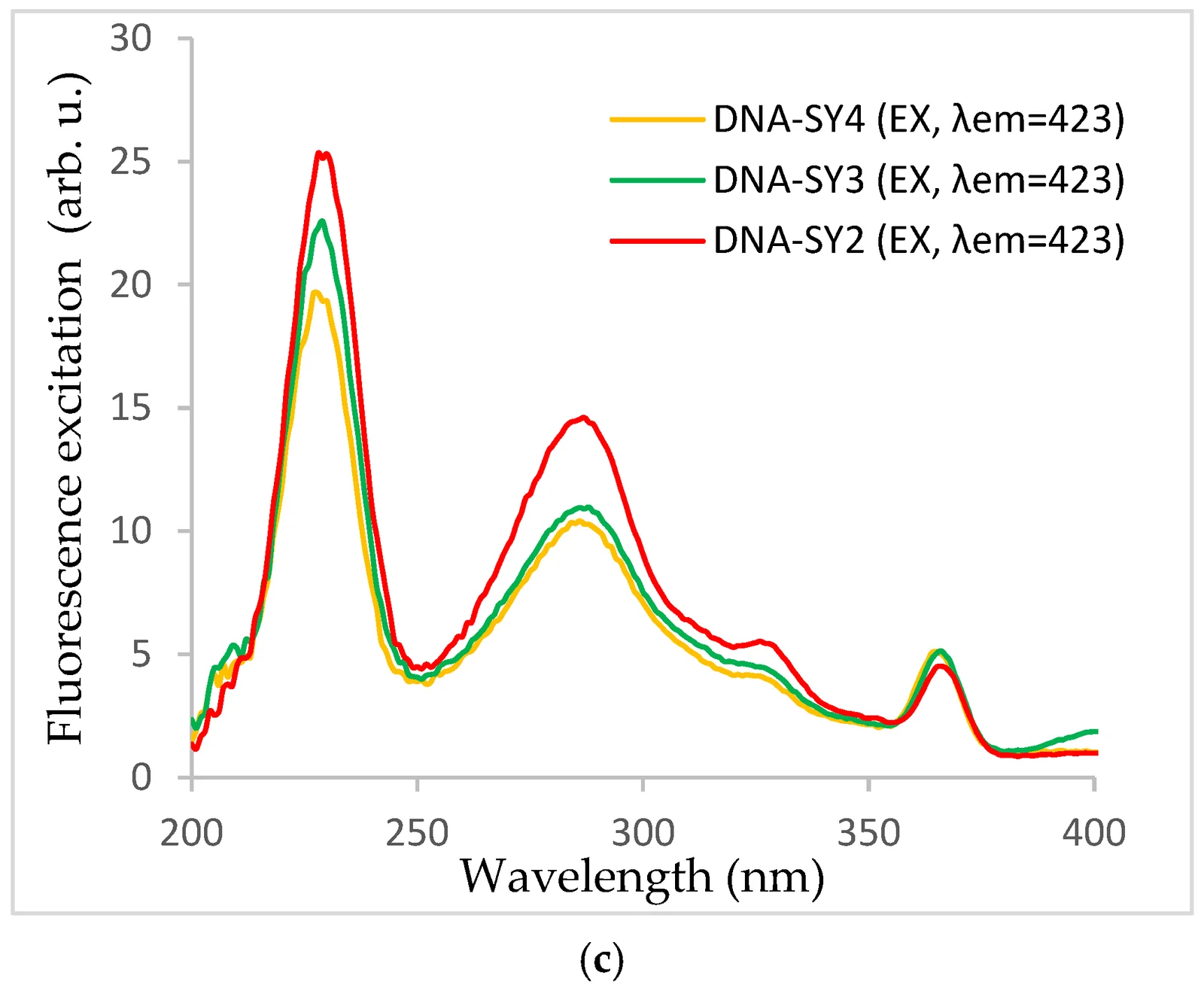
Fluorescence emission spectrum of aqueous SY solutions (**a**) and fluorescence excitation spectra of aqueous SY solutions (**b**) and the DNA-SY system (**c**) at different SY concentrations.

The emission and excitation spectra of aqueous solution of sunset yellow are shown in Figure 7a and Figure 7b, respectively. At a concentration of 10^−4^ M, the Stokes shift is 148 nm. For a concentration of 2 × 10^−5^ M, the shift is 151 nm, while at 10^−5^ M, the Stokes shift is 147 nm. Figure 7c shows the excitation spectra of DNA-SY complexes at different concentrations, recorded at the emission wavelength of 423 nm. A decrease in fluorescence intensity is observed as the SY concentration decreases.

**Figure 8:**
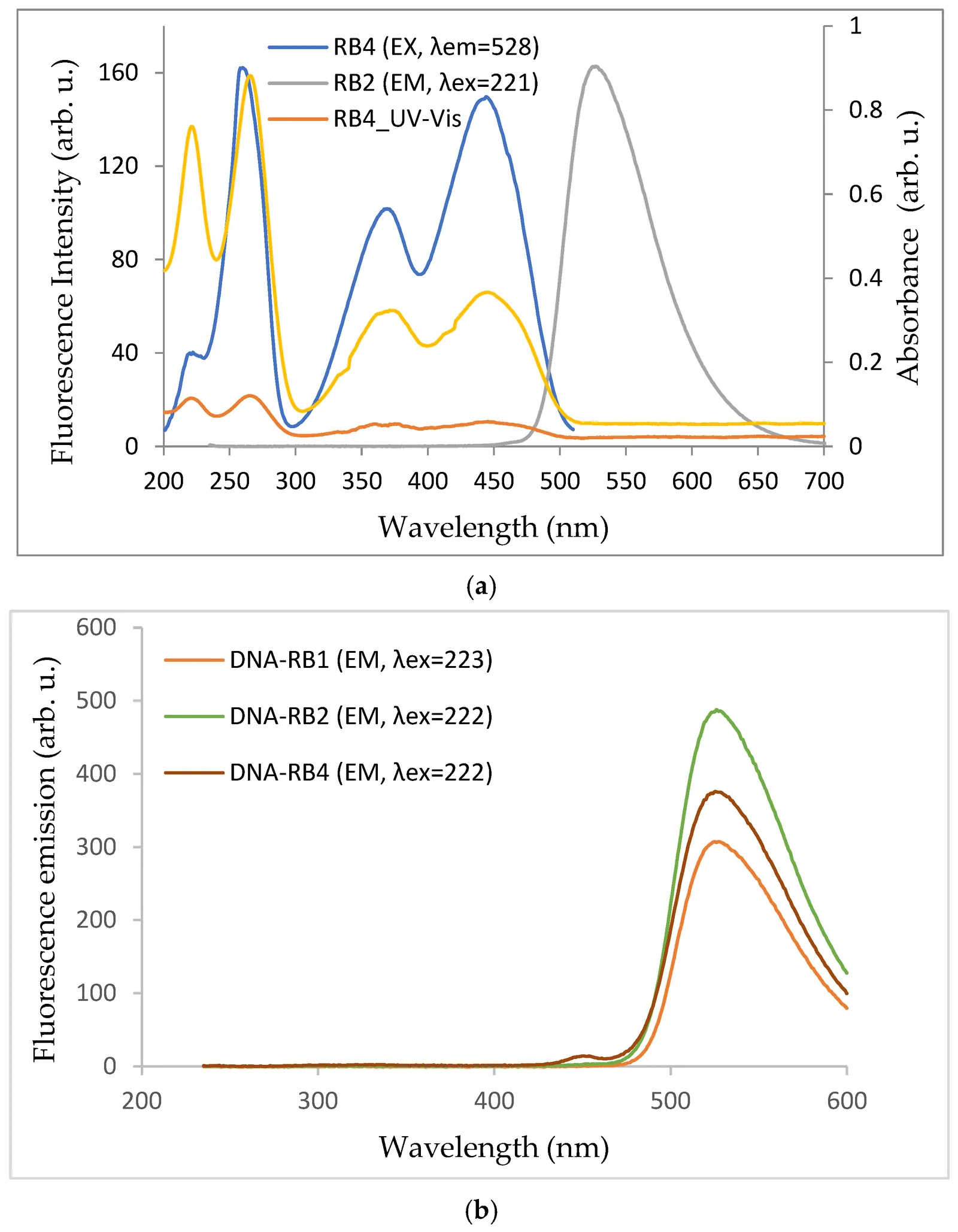

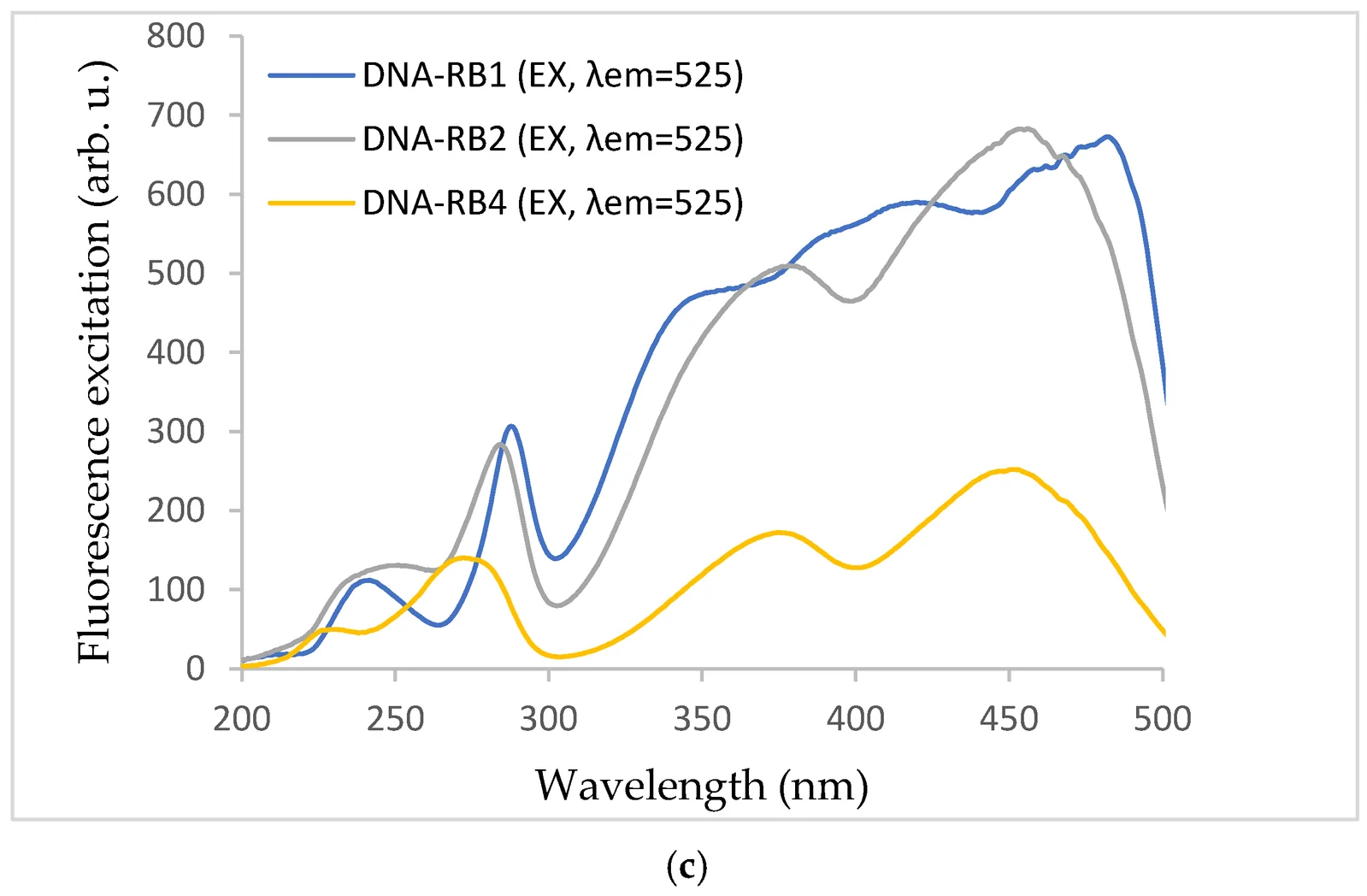
Photoluminescence spectra of RB (**a**) and DNA-RB solutions (**b**,**c**) at different RB concentrations.

Figure 8 displays the fluorescence spectra of RB (a) and DNA functionalized with RB (b, c). A decrease in fluorescence intensity is observed as the RB concentration decreases when DNA is functionalized with RB.

### 3.3. FTIR

**Figure 9:**
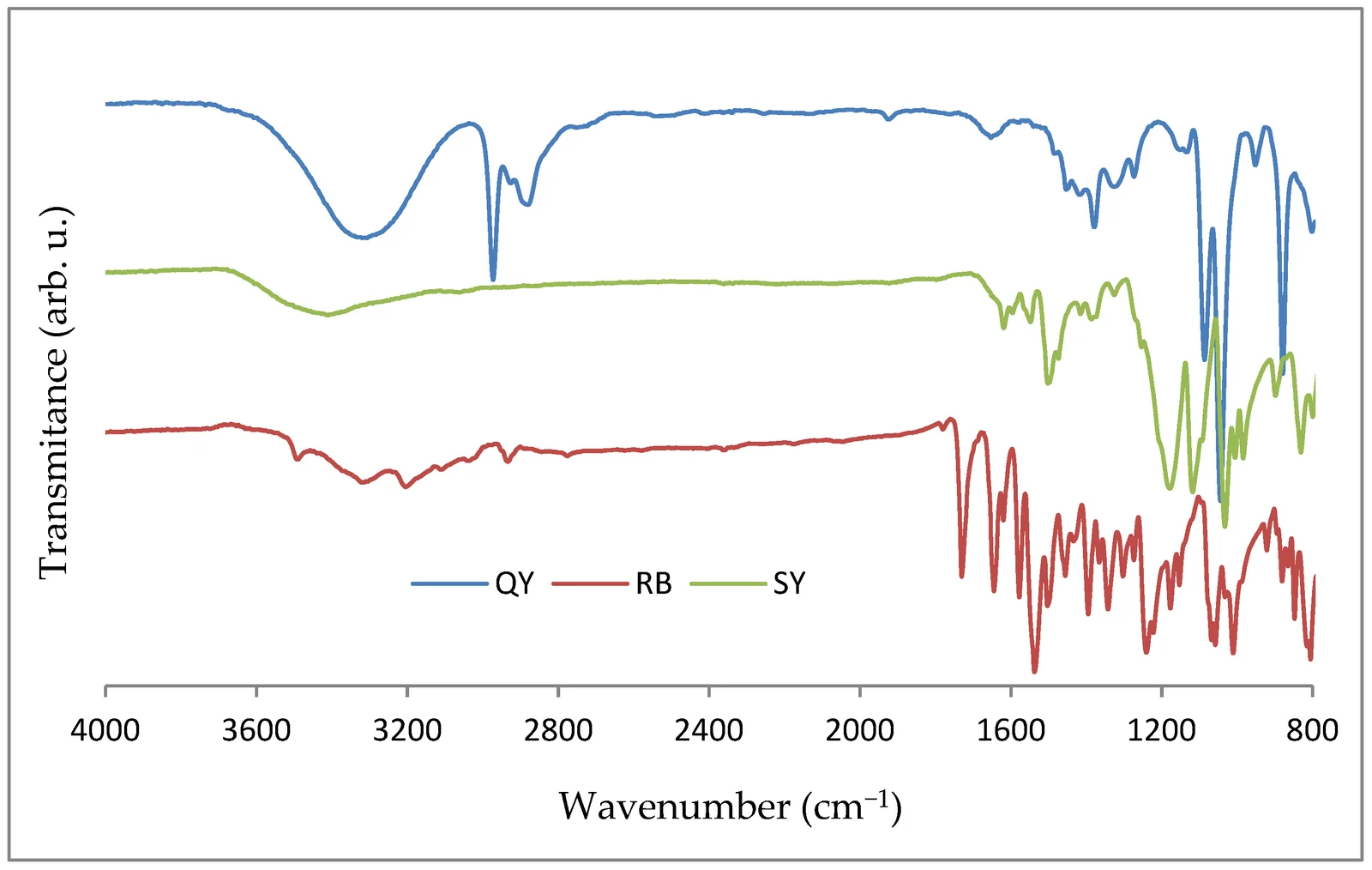
FTIR spectra of QY, SY and RB solutions.

As shown in Figure 9, several characteristic bands were observed in the FTIR spectrum of quinoline yellow (QY). The broad band at 3307.8 cm^−1^ can be attributed to O–H stretching vibrations, most likely associated with adsorbed water. The bands at 2972.7 and 2880.3 cm^−1^ can be assigned to C–H stretching vibrations. The bands in the region around 1600–1700 cm^−1^ can be associated with the C=O and aromatic/quinoline C=C/C=N vibrations of the quinophthalone structure. The bands observed in the region of approximately 1000–1250 cm^−1^ can be attributed mainly to vibrations of the sulfonate (SO_3_^−^) groups. The band at 878.9 cm^−1^ can be associated with out-of-plane C–H bending vibrations of the aromatic system. Overall, the observed FTIR bands are consistent with the presence of carbonyl, sulfonate, aromatic, and quinoline functionalities in quinoline yellow [20].

In the case of SY, the IR vibrations were identified at the following wavenumbers: 3410.9 cm^−1^, assigned to the vibration of the O-H and -C-H bonds; 1619.1 cm^−1^, corresponding to the C=O vibration; in-plane O-H bending vibration and stretching vibration of the benzene and naphthalene rings; 1548.1 cm^−1^, attributed to the in-plane O-H bending vibration and stretching vibration of C-H bonds in the naphthalene ring; 1501.7 cm^−1^, corresponding to the C=C vibration in the benzene ring and vibrations arising from the coupling between the naphthalene ring and the asymmetric stretching vibrations of the azo group; 1386.5 cm^−1^, assigned to the stretching vibration of the azo group (–N=N–) coupled with the benzene ring; 1177.8 cm^−1^, corresponding to the vibration of the SO_3_ group and the vibration of the C-N bond; 1118.2 cm^−1^, assigned to the symmetric stretching vibration of the C-N bond; 1032.7 cm^−1^, corresponding to the symmetric stretching vibration of the S=O bond in the SO_3_-H group; 1005.5 cm^−1^, attributed to the S=O bond stretching vibration; 984.3 cm^−1^, corresponding to the out-of-plane bending vibration of the C-H bonds in the naphthalene ring; 898.5 cm^−1^, assigned to the in-plane bending vibration of the C-N=N-C chromophore group coupled with bending vibrations of the benzene and naphthalene rings; and 830.9 cm^−1^, corresponding to the out-of-plane O-H bending vibration [27,28].

A similar infrared analysis of the RB solution reveals stretching vibrations at 3502, 3344, and 3204.1 cm^−1^, which are characteristic of the -N-H and -O-H groups, respectively, as well as vibrations associated with intramolecular hydrogen bonding in the –OH groups. The band at 2942 cm^−1^ can be attributed to the asymmetric stretching vibrations of the C-H bonds in the methylene side groups. At 1730.3 and 1644.9 cm^−1^, vibrations characteristic of the -C=O and -C=N- groups, respectively, were observed. The band at 1577.7 cm^−1^ was assigned to asymmetric C=C stretching vibrations in aromatic rings, while the band at 1395.0 cm^−1^ was attributed to the symmetric deformation vibrations of the CH_3_ groups. The remaining bands were assigned as follows: 1342.4 cm^−1^ to C-C stretching vibrations; 1240.8 cm^−1^ to O-H bond deformation vibrations; 1177.2 cm^−1^ to symmetric C-N stretching vibration; 1153.2 cm^−1^ to symmetric C-C stretching vibrations; 1057.9 and 1010.6 cm^−1^ to C-O stretching vibrations; and 921.5 and 847.9 cm^−1^ to out-of-plane bending vibrations of the C-H bonds in the benzene ring [29].

### 3.4. Extraction of Light-Induced Phase Modulation and Determination of the Nonlinear Refractive Index

**Figure 10:**
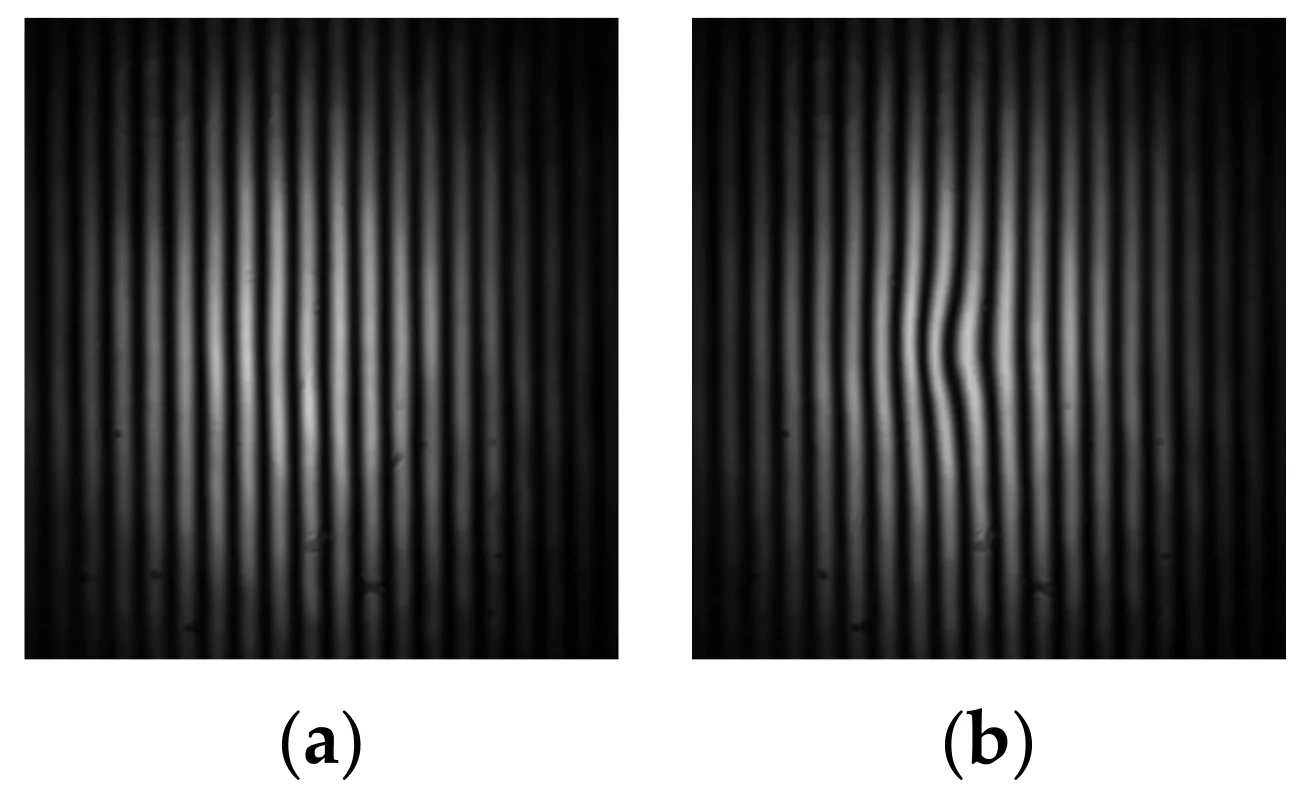
A set of fringes recorded with the pump beam OFF (**a**) and ON (**b**), respectively. (**a**) $∆\Phi pumpOFF\left(x,y\right)=\Delta \Phi_{tilt}\left(x,y\right)$; (**b**) $\Delta \Phi pumpON\left(x,y\right)=\Delta \Phi_{nl}\left(x,y\right)+\Delta \Phi_{tilt}\left(x,y\right)$.

The interference pattern generated by the two He-Ne laser beams on the photosensitive matrix of the LBA contains information about the phase difference (∆Φ) between the beams propagating along the two arms of the interferometer. When the pump beam is turned OFF, the phase difference is due only to the tilt between the two interfering beams, ∆Φ_tilt_. If the pump beam is turned ON, the phase difference has two components: one due to the tilt and the other due to the light-induced phase modulation, ∆Φ_NL_. Since the spot size of the test beam is larger than that of the pump beam focused onto the nonlinear sample plane, the interference pattern formed by parallel and equidistant fringes becomes locally distorted in the region corresponding to the pump beam spot. Fringe patterns recorded under these two conditions are shown in Figure 10.

The converging lenses L2 and L3, having the same focal length, are positioned in the interferometric configuration at equal distances from the LBA. This arrangement ensures that both beams reach the LBA plane with the same wavefront curvature, producing a pattern of parallel and equidistant fringes in the absence of pump beam excitation. Consequently, the area of distorted fringes in the interference pattern corresponds to the region in which the optical properties of the sample are modified by the pump beam.

The phase modulation induced by the pump beam in the investigated nonlinear optical samples is obtained by processing the interference fringe patterns recorded with the pump beam ON and OFF, alternately. The phase information contained in the experimental fringe patterns is extracted using a method developed by us [14,15,16], based on Fourier analysis [30,31,32], for Direct Spatial Reconstruction of Optical Phase (DSROP) [28,29]. The DSROP method, described in detail in [14,15,16], is based on the Fourier transform. A specific feature of this method is that the phase information can be extracted from a single fringe pattern.

From the fringe pattern recorded with the pump beam OFF, we extract the phase distribution ∆Φ*_PumpOFF_*. From the fringe pattern recorded with the pump beam ON, we obtain the phase distribution ∆Φ*_PumpON_*. By subtracting ∆Φ*_PumpOFF_* from ∆Φ*_PumpON_*, we obtain the spatial distribution of the phase modulation induced by the pump beam.

**Figure 11:**
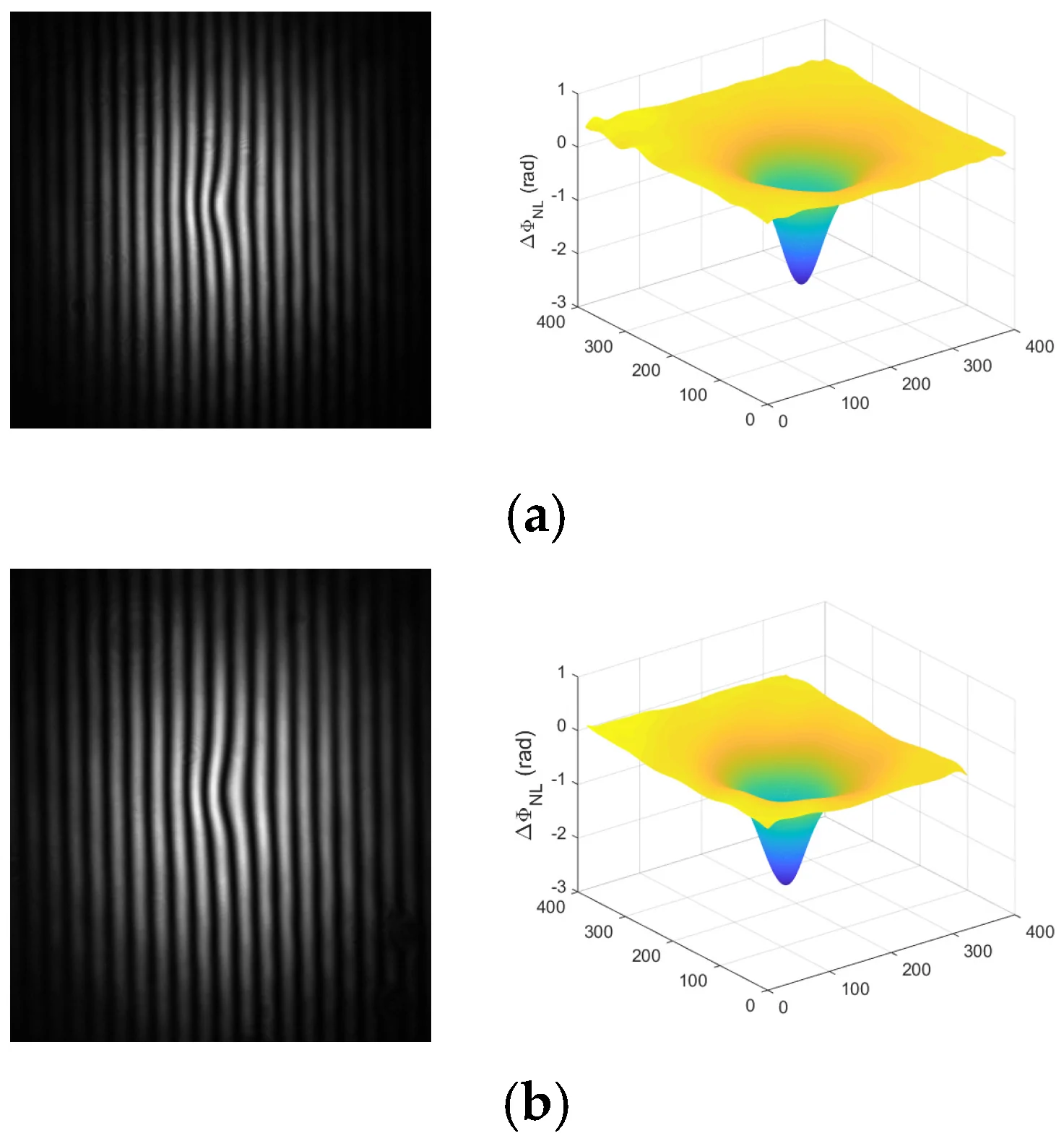

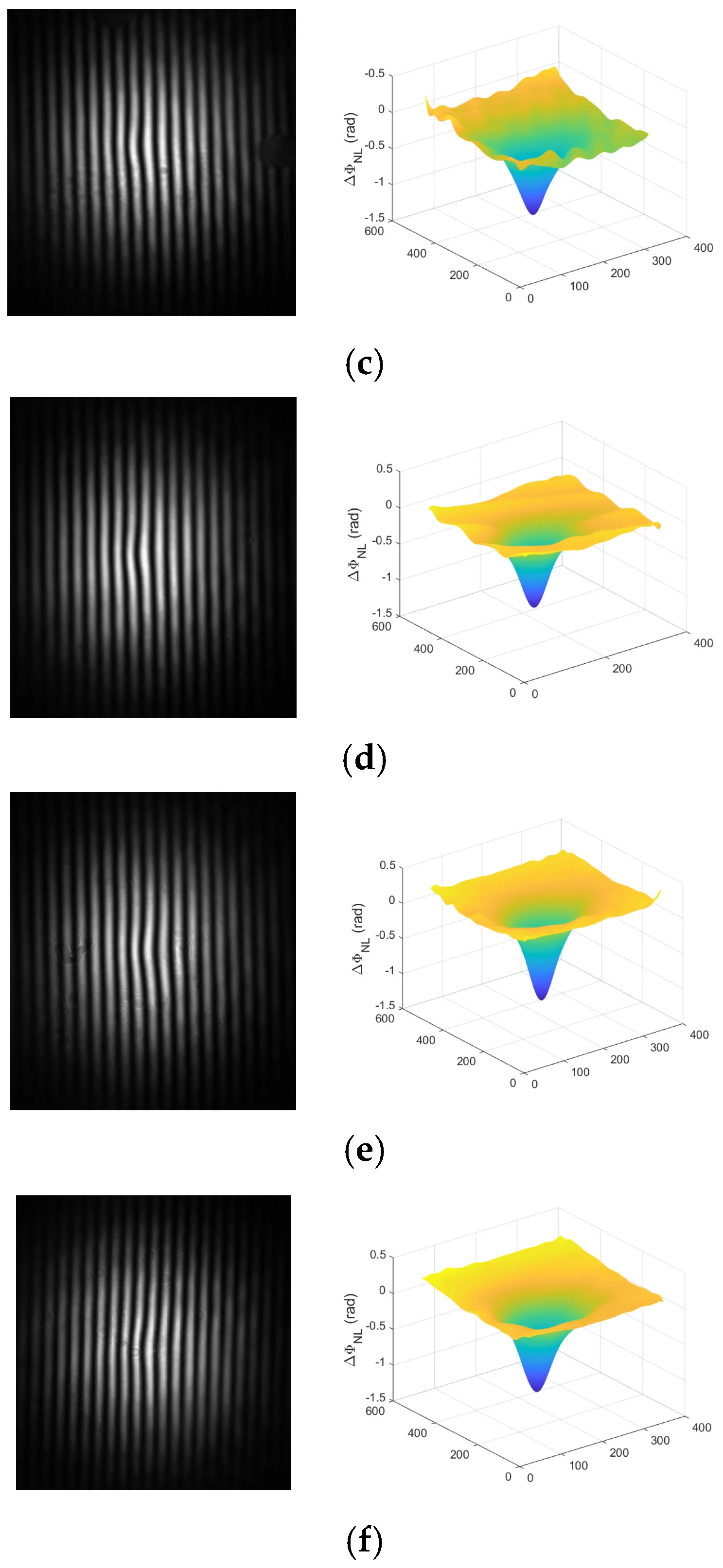
Locally distorted fringe patterns and their corresponding 3D light-induced phase modulation computed from these patterns using the DSROP method. (**a**) DNA-QY, ∆Φ_NL_ (rad) = −2.75; (**b**) QY, ∆Φ_NL_ (rad) = −2.18; (**c**) DNA-SY, ∆Φ_NL_ (rad) = −1.51; (**d**) SY, ∆Φ_NL_ (rad) = −1.28; (**e**) DNA-RB, ∆Φ_NL_ (rad) = −1.64; (**f**) RB, ∆Φ_NL_ (rad) = −1.25.

By processing fringe patterns such as those shown in Figure 10a,b corresponding to the investigated samples, the phase distributions ∆Φ*_PumpOFF_* and ∆Φ*_PumpON_* are calculated following the procedure described in detail in [16,31,32], from which the corresponding light-induced phase modulation ∆Φ*_NL_* is determined. From the spatial phase modulation distribution as a function of the excitation intensity, we determine the change in the refractive index induced in the sample and the corresponding nonlinear refractive index (*n*_2_). Measurements were performed in the intensity range of 0.35 ÷ 1.63 W/cm^2^ for each solution sample. A set of phase maps obtained at the highest excitation intensity for the investigated samples is presented in Figure 11.

The analysis of these images reveals that the modulation of the intensity distribution of the test beam increases with increasing intensity of the excitation beam.

Due to the high absorption in the sample, the excitation intensity, *I*_pump_, decreases as it propagates through the sample according to the Lambert–Beer law, $I_{pump}(z)=I_{pump}(0)\exp(-\alpha z)$, which leads to a *z*-dependence of the refractive index modulation of the same form, $\Delta n\left(z\right)=n_{2}I_{pump}(z)=\Delta n\left(0\right)\exp(-\alpha z)$. The maximum refractive index modulation, ∆*n*(0) = *n*_2_*I*_pump_(0), occurs at the entrance face of the sample, z = 0, where the excitation laser beam enters the sample. From the phase modulation of the sample beam,$\Delta \Phi_{NL}\left(L\right)$, as it propagates through the sample with a refractive index modified by the excitation beam, $\Delta \Phi_{NL}\left(L\right)=kn_{2}I_{pump}(0)L_{eff}=k\Delta n(0)L_{eff}$ (*k*—wave number corresponding to the wavelength of the sample beam), $\Delta n(0)=\Delta \Phi_{NL}\left(L\right)/\left(kL_{eff}\right)$ the nonlinear refractive index, *n*_2_, associated with the processes induced in the sample by optical excitation, can also be determined: $n_{2}=\Delta \Phi_{NL}\left(L\right)/\left(kI_{pump}\left(0\right)L_{eff}\right)$.

Table 2 summarizes the results obtained from the analysis of the experimental dependence of the spatial phase modulation distribution on the intensity of the excitation laser beam, including the spatial phase modulation, ΔΦ_NL_, as a function of the excitation laser intensity; the maximum refractive index modulation, ∆n; and the nonlinear refractive index, n_2_.

Analysis of the data presented in Table 2 shows that the nonlinear optical parameters obtained for the DNA-containing solutions are higher in magnitude than those obtained for the corresponding dye solutions without DNA. These results indicate that the presence of DNA is associated with an enhancement of the observed all-optical spatial phase modulation under the experimental conditions investigated.

**Figure 12:**
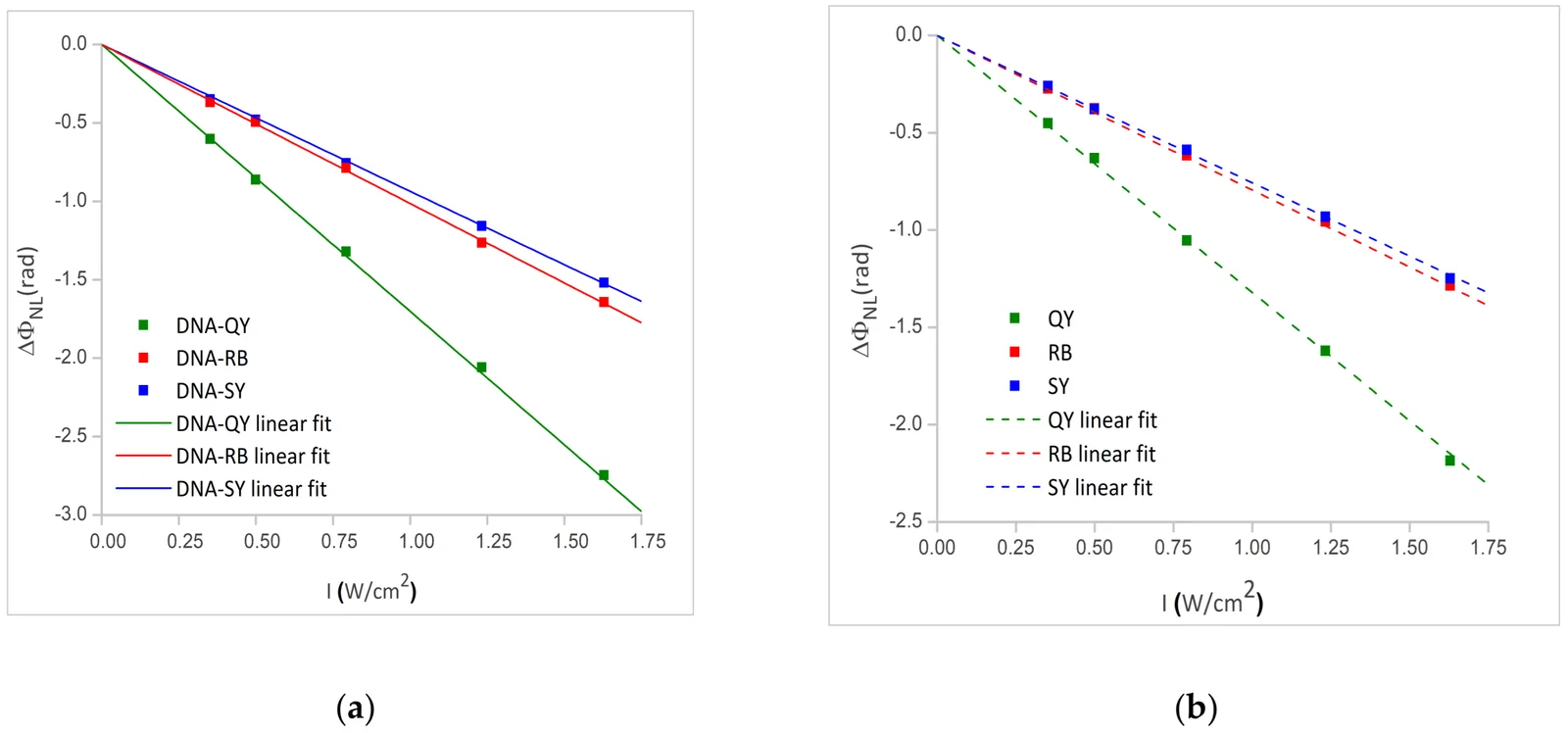
Dependence of the phase change on the intensity of the excitation beam in (**a**) DNA-QY, DNA-SY, DNA-RB and (**b**) QY, SY, RB solutions.

The dependence of the phase-modulation magnitude on the pump intensity, determined from the experimental data, is shown in Figure 12. The negative sign of the phase modulation, ΔΦ_NL_, corresponds to the formation of a divergent lens in the sample induced by the excitation beam.

In the range of pump intensities used in our experiments, the dependence of the phase modulation, ∆Φ_NL_, on the excitation intensity, *I*, is well fitted by the linear one given in Table 3.

In the intensity range considered in Figure 12, where the phase modulation is linear, it is also reversible. The experimental results reveal the different responses of the three samples to the light excitation, as evidenced by different slopes of the phase-modulation dependence on the excitation-beam intensity, highlighting the beneficial influence of DNA. In the experiments performed on the investigated solutions, the phase modulation was determined after reaching the stationary state, i.e., a few seconds after the onset of optical excitation.

## 4. Conclusions

The performed UV-Vis spectroscopy shows that DNA, due to its conjugated π structure, as well as QY, with its specific electronic transitions, has optical characteristics that make them suitable for use in developing eco-friendly and efficient optoelectronic materials and devices. The analysis of the absorption spectra of SY highlights the presence of characteristic peaks at 234, 311, and around 483 nm, which correspond to the π-π* electronic transitions of the aromatic rings and the azo bond transitions in the compound’s structure. Riboflavin shows electronic transitions clearly attributed to n-π* and π-π* values, which confirms the stability of the aromatic structures and the functional groups involved.

The comparative study of the fluorescent response of the food colorants quinoline yellow (QY), sunset yellow (SY), and riboflavin (RB), incorporated into DNA matrices, highlights the potential of these materials for integrated photonics applications. QY shows a Stokes shift of 62 nm, enough to avoid spectral overlap between the excitation and emission wavelengths. SY shows a large Stokes shift, suitable for photonic applications, while RB embedded in DNA keeps its fluorescent properties, and its emission intensity decreases proportionally with the reduction in RB concentration. This linear dependence indicates a stable and predictable interaction between RB and DNA, making it suitable for precise applications in optical sensors and other photonic devices.

The light-induced nonlinear responses in QY/DNA-QY, SY/DNA-SY, and RB/DNA-RB solutions were investigated using a pump–probe interferometric method, in which the optical phase information is extracted from a single fringe pattern (DSROP algorithm). Thus, we determined the 3D spatial distribution, sign and magnitude dependence of the light-induced phase modulation. From these, we computed the phase modulation ΔΦ_NL_, the maximum refractive index modulation ∆*n* and the nonlinear refractive index *n*_2_. The results reveal distinct responses among the studied samples. The observed differences among the systems suggest that both the molecular structure of the dye and its interaction with the DNA matrix play an important role in determining their nonlinear optical behavior. Among the studied samples, DNA-QY, QY exhibited the strongest nonlinear response, followed by DNA-RB/RB and DNA-SY/SY. The DNA-QY, QY system is a promising candidate for applications involving nonlinear optical modulation, optical switching, signal processing, and other photonic devices requiring materials with enhanced nonlinear optical properties.

## Figures and Tables

**Table 1:** Composition and concentrations of the prepared solutions.

Sample	QY	SY	RB
		Concentration (M)	
DNA-QY1	10^−4^	-	-
DNA-QY2	5 × 10^−5^	-	-
DNA-QY3	2 × 10^−5^	-	-
DNA-QY4	10^−5^	-	-
DNA-SY1	-	10^−4^	-
DNA-SY2	-	5 × 10^−5^	-
DNA-SY3	-	2 × 10^−5^	-
DNA-SY4	-	10^−5^	-
DNA-RB1	-	-	10^−4^
DNA-RB2	-	-	5 × 10^−5^
DNA-RB3	-	-	2 × 10^−5^
DNA-RB4	-	-	10^−5^

**Table 2:** The phase modulation ΔΦ_NL_, maximum refractive index modulation ∆n and the nonlinear refractive index n_2_, determined from the fit of the experimental data.

**Sample**	**DNA-QY/QY**		
Intensity (W/cm^2^)	∆Φ_NL_ (rad)	∆n × 10^−4^	n_2_ × 10^−4^ (cm^2^/W)
1.63	−2.75/−2.18	−5.53/−4.40	−3.41/−2.62
1.23	−2.06/−1.62	−4.15/−3.26	−3.39/−2.61
0.80	−1.32/−1.06	−2.66/−2.12	−3.38/−2.60
0.50	−0.86/−0.63	−1.74/−1.27	−3.38/−2.60
0.35	−0.60/−0.45	−1.22/−0.91	−3.37/−2.59
**Sample**	**DNA-SY/SY**		
Intensity (W/cm^2^)	∆Φ_NL_ (rad)	∆n × 10^−4^	n_2_ × 10^−4^ (cm^2^/W)
1.63	−1.51/−1.28	−3.06/−2.59	−2.00 /−1.59
1.23	−1.16/−0.95	−2.33/−1.93	−1.94 /−1.58
0.80	−0.76/−0.62	−1.53/−1.24	−1.93 /−1.58
0.50	−0.48/−0.38	−0.97/−0.75	−1.92 /−1.57
0.35	−0.35/−0.27	−0.70/−0.55	−1.91/−1.56
**Sample**	**DNA-RB/RB**		
Intensity (W/cm^2^)	∆Φ_NL_ (rad)	∆n	n_2_ × 10^−4^ (cm^2^/W)
1.63	−1.64/−1.25	−3.22/−2.41	−2.10/−1.53
1.23	−1.26/−0.93	−2.47/−1.88	−2.05/−1.52
0.80	−0.79/−0.59	−1.59/−1.19	−2.01/−1.52
0.50	−0.49/−0.38	−0.99/−0.76	−1.99/−1.50
0.35	−0.37/−0.26	−0.74/−0.52	−1.98/−1.49

**Table 3:** Linear fitting parameters of the dependence of the phase modulation, ∆Φ_NL_, on the excitation intensity, I.

Sample	∆Φ_NL_ (Rad)
DNA-QY	$\Delta \Phi_{NL}(rad)=-1.70\cdot \left(\frac{rad}{W/cm^{2}}\right)\cdot I\left(W/cm^{2}\right)$
DNA-RB	$\Delta \Phi_{NL}(rad)=-1.013\cdot \left(\frac{rad}{W/cm^{2}}\right)\cdot I\left(W/cm^{2}\right)$
DNA-SY	$\Delta \Phi_{NL}\left(rad\right)=-0.936\cdot \left(\frac{rad}{W/cm^{2}}\right)\cdot I\left(W/cm^{2}\right)$
QY	$\Delta \Phi_{NL}(rad)=-1.320\cdot \left(\frac{rad}{W/cm^{2}}\right)\cdot I\left(W/cm^{2}\right)$
RB	$\Delta \Phi_{NL}\left(rad\right)=-0.793\cdot \left(\frac{rad}{W/cm^{2}}\right)\cdot I\left(W/cm^{2}\right)$
SY	$\Delta \Phi_{NL}(rad)=-0.756\cdot \left(\frac{rad}{W/cm^{2}}\right)\cdot I\left(W/cm^{2}\right)$

## Data Availability

The original contributions presented in this study are included in the article. Further inquiries can be directed to the corresponding author.
